# STYK1/NOK affects cell cycle late mitosis and directly interacts with anaphase-promoting complex activator CDH1

**DOI:** 10.1016/j.heliyon.2022.e12058

**Published:** 2022-12-05

**Authors:** Su-Lin Zeng, Suraj S. Patel, Mo-Qi Lv, Daphne Zhu, Wen H. Shen, Li Liu

**Affiliations:** a Department of Pathogen Biology, Institute of Basic Medical Sciences, Chinese Academy of Medical Sciences & School of Basic Medicine, Peking Union Medical College, Beijing 100005, China; b Department of Radiation Oncology, Weill Cornell Medicine, New York, New York 10065, USA

**Keywords:** Cell cycle, STYK1/NOK, APC/C, Protein-protein interaction, CDH1

## Abstract

The novel oncogene STYK1/NOK plays critical roles in cancer development. However, its regulation during cell division is less defined. In this paper, we show that over-expression of STYK1/NOK caused mitotic arrest and cytokinesis defects. The protein level of STYK/NOK fluctuated during the cell cycle, with a peak at mitosis and a quick reduction upon mitotic exit. The cell cycle-related expression pattern of STYK1/NOK resembled the one of aurora kinases and polo-like kinase 1. Depletion of APC3 led to accumulation of STYK1/NOK and to the G2/M arrest. Co-immunoprecipitation experiment demonstrated the direct interaction of STYK1/NOK with CDH1. Overexpression of CDH1 shortened the half-life of STYK1/NOK. The kinase domain, but not the five D boxes, of STYK1/NOK was responsible for the interaction with CDH1. Altogether, our data demonstrated for the first time that STYK1/NOK could affect cell division, probably by directly targeting key components of APC/C such as CDH1 at late mitosis. Current study may provide a vital mechanistic clue for understanding the roles of STYK1/NOK in mitosis and cytokinesis during STYK1NOK mediated genomic instability and oncogenesis.

## 1 Introduction

Anaphase-promoting complex or cyclosome (APC/C) is an E3 ligase composed of at least 14 subunits. It plays an important role in regulating the separation of sister chromatids during mitosis [1, 2]. Among these 14 subunits, APC3 is one of the five tetratricopeptide repeat (TPR) subunits that mediate CDC20 homolog 1 (CDH1) binding [3]. Both CDH1 and cell division cycle protein 20 homolog (CDC20) are well-known co-activators that activate and recruit substrates for the E3 ligase APC/C [4]. Both CDC20 and CDH1 contain a Trp-Asp 40 (WD-40)-repeats domain in their carboxyl terminuses [1], which can specifically recognize and interact with degrons, such as destruction box (D-box) and KEN-box in the substrates [5]. The functions of APC/C associated with CDC20 or CDH1 are regulated spatiotemporally during cell cycle [2, 6]. In early mitosis, cyclin-dependent kinase 1 (CDK1) exhibits a high activity to phosphorylate both CDC20 and APC/C. The activated CDC20-bound APC/C (APC/C^CDC20^) ubiquitinates cyclin A and Nek2A [1], and subsequently reduces the activity of CDK1 [7]. CDC20 is dephosphorylated as the cells progress toward anaphase, and when all the chromosomes display on the metaphase plate, APC/C mediates the proteolysis of cyclin B and securin to induce anaphase onset. Subsequently, the reduced activity of CDK1 lifts the inhibition on CDH1, permitting CDH1 activation and association with APC/C. APC/C^CDH1^ then degrades multiple substrates including aurora kinases A and B, allowing the separation of sister chromatids to trigger mitotic exit. The normal function of APC/C^CDH1^ ensures proper separation of sister chromatids to maintain the integrity and stability of genome. CDH1-null cells exhibited chromosome breaks and aneuploidy [8]. As integral components of the functional APC/C^CDH1^ E3 ligase complex, its enzymatic substrates also play critical roles in maintaining genome stability. For example, overexpression of aurora A and securin has been shown to cause cytokinesis failure, leading to genomic instability [9].

STYK1/NOK is a single member family within the receptor protein tyrosine kinase superfamily [10]. It is initially believed to be a pseudo kinase due to its lack of a Asp-Phe-GlyIt (DFG) motif, which is important for γ-phosphate transfer from ATP [11]. Despite of this defect, functional study showed that STYK1/NOK could act as a potent oncogene to provoke tumorigenesis and metastasis in nude mice [12]. High levels of STYK1/NOK expression have been reported in many types of cancers including leukemia [13], lung cancer [14], breast cancer [15] and colorectal cancer etc [16]. However, the mechanism by which STYK1/NOK promotes tumorigenesis and metastasis, and its role in cell cycle progression remain largely elusive. Here we reported that STYK1/NOK was a cell-cycle regulated protein that peaked in mitosis. Overexpression of STYK1/NOK led to mitotic arrest and prolonged cytokinesis. Mechanistic evidence showed that STYK1/NOK could directly interact with the key components of APC/C complex such as CDH1. These data provided mechanistic insights into the roles of STYK1/NOK during the cell cycle progress especially at stage of late mitosis, which could be critical for STYK1/NOK mediated genomic instability and oncogenesis.

## 2 Methods

### 2.1 Cell lines, stable cell constructions and antibodies

Human prostatic adenocarcinoma cell line PC-3, human brain glioblastoma cell line U-87 MG, human colorectal adenocarcinoma cell line DLD-1, human cervical cancer cell line HeLa, human embryonic kidney 293 derived cell line HEK293T, hTERT-immortalized retinal pigment epithelial cell line RPE-1 and human colorectal cancer cell line HCT116 were from the American Type Culture Collection. All the cell lines were cultured in MEM (Corning, Manassas, VA, USA) containing 8% FBS (Atlanta biologicals, Flowery Branch, GA, USA). Genetically modified stable cell lines were established according to the protocol described previously [17]. The pTRIPZ plasmid was a gift from Dr. Sandra Demaria at Weill Cornell Medicine. The packaging plasmids (pPAX2, pMD2 and pTRIPZ) were co-transfected into HEK293T cells for the production of recombinant lentiviruses. Virus-containing conditional media were collected 48 h after transfection and used to infect HeLa or DLD-1 cells. Infected HeLa and DLD-1 cells were selected with 1 μg/mL and 2 μg/mL of puromycin, respectively, for more than 5 days. The efficiency of virus infection was evaluated with a TurboRed reporter via flow cytometry. More than 90% cells were confirmed positive in all the infected cell lines. STYK1/NOK antibody was obtained from Proteintech (Wuhan, China). Aurora A and aurora B antibodies were obtained from Cell Signaling Technology (Danvers, MA, USA). PLK1 and second antibodies were obtained from Santa Cruz Biotechnology (Dallas, TX, USA). GAPDH antibody was obtained from Medical & Biological Laboratories (Woburn, MA, USA).

### 2.2 Constructions of expression plasmids and transfection

The pTRIPZ vector with a tetracycline-inducible promoter was used to construct shRNAs that target APC3 or STYK1. XhoI and EcoRI restriction sites were used to clone shRNAs. The oligo sequences used for the constructions of APC3 and STYK1 specific shRNAs are: shAPC3: ACCAAAAGAGCCTTAGTTTAAA and shSTYK1: CGCCTAGAAGCTGCCATTAAAA, respectively. STYK1/NOK cDNAs were amplified from PC-3 cells and sub-cloned into HindIII and XbaI sites of pcDNA3.0 to construct pcDNA3.0-STYK1/NOK. STYK1/NOK cDNAs were also sub-cloned into AgeI and MluI sites of pTRIPZ to generate pTRIPZ-STYK1/NOK. The D-box mutants of STYK1/NOK were created by a site-directed mutagenesis approach using In-Fusion® Snap Assembly Master Mix kit from Takara following the manufacturer’s instructions. The primers for the five D-box mutants of STYK1/NOK were:

△D1 Forward: CCAAGCTTATGGGCATGACAGAATGCAGTCTCAGTGACAA
△D1 Reverse: TTGTCACTGAGACTGCATTCTGTCATGCCCATAAGCTTGG
△D2 Forward: TGGCTAAGCTGCAGGTGCCGTCTGAAGTTCTGGAGCAGAT
△D2 Reverse: ATCTGCTCCAGAACTTCAGACGGCACCTGCAGCTTAGCCA
△D3 Forward: TCCATGGGGATGTGGCAGCCATGCAAAGTGATCTCACTGC
△D3 Reverse: GCAGTGAGATCACTTTGCATGGCTGCCACATCCCCATGGA
△D4 Forward: TCAAGTGGCTTGCCCCAGAAAGACCTGCTAGCATCAGAGC
△D4 Reverse: GCTCTGATGCTAGCAGGTCTTTCTGGGGCAAGCCACTTGA
△D5 Forward: GCCCCTCACCTAGAGAGCTGGAAGCTGCCATTAAAACTGC
△D5 Reverse: GCAGTTTTAATGGCAGCTTCCAGCTCTCTAGGTGAGGGGC

pcDNA3.0-STYK1/NOK was used as a template.

CDH1 cDNAs were sub-cloned into EcoRI and NotI sites of the expression vector containing FLAG tag, which was a gift from Dr. W Gu [18] to generate the construct of FLAG-CDH1. Cells were transfected with plasmids which were mixed with 100-fold volume of opti-MEM (v/w) and 8-fold volume of PEI (v/w) and cell extraction was performed 48 h post transfection.

### 2.3 Cell cycle synchronization and flow cytometry

In order to synchronize the cells at G1/S boundary, the cells were first treated with 2 mM thymidine for 18 h, followed by 9 h release and then subsequently treated with 2 mM thymidine for additional 15 h. For synchronization at G2/M boundary, cells were first treated as described above and after the second thymidine block, the cells were released by incubating in a fresh medium for 2 h, and then treated with 10 μM RO3306 for additional 10 h prior to harvesting. Synchronized cells were fixed with 4% paraformaldehyde (Electron Microscopy Sciences, Hatfield, PA, USA) in PBS, then resuspended in 1 μg/mL of DAPI solution and subjected to flow cytometry analysis using MACSQuant® Analyzer 10,000 to 40,000 cells were detected in each sample. Data were analyzed using FlowJo software (Tree Star).

### 2.4 Cycloheximide chase assay

To investigate the turnover of exogenous STYK1/NOK, cells with ectopic STYK1/NOK and CDH1 expressions were treated with 50 μg/mL of cycloheximide for different time durations before harvesting. Total protein extracts were prepared, resolved in SDS-PAGE gel and immunoblotted with anti-STYK1/NOK and anti-GAPDH antibodies. The band intensity of STYK1/NOK was quantified using FIJI software, and the relative levels of STYK1/NOK at each time point were normalized to GAPDH. Time 0 h was set to 100%, and other time points were normalized to time 0 h. The protein half-life (t_1/2_) was calculated according to the fit curve.

### 2.5 Immunoblotting and co-immunoprecipitation

For immunoblotting, cells were lysed in RIPA buffer (150 mM NaCl, 1% Triton X-100, 0.5% sodium deoxycholate, 0.1% SDS, 50 mM Tris-HCl, pH7.6) supplemented with protease inhibitors. Protein concentration was measured by Bradford assay (Alfa Aesar, Ward Hill, MA, USA). About 15 μg of total proteins was resolved on SAS-PAGE gel. The reaction product was transferred onto a nitrocellulose membrane and subjected to immunoblotting analysis. For co-immunoprecipitation, the cells were first treated with 10 μM MG132 for 4 h before harvesting. Protein lysates (about 2000 μg) were mixed with 20 μL of anti-FLAG affinity gel (Sigma, Burlington, MA, USA) at 4 °C overnight. Then, the affinity gel was collected and washed with 1xPBS for 6 times (5 min each time), and resuspended with SDS loading buffer and boiled for 10 min. The reaction products were resolved onto a 12% SDS-PAGE, transferred to a nitrocellulose membrane and subjected to immunoblotting analysis. A fraction of protein lysate without immunoprecipitation was loaded as input.

### 2.6 Immunofluorescence and microscopy

Immunofluorescence was performed according to the protocol described previously [19]. Briefly, cells were fixed with 70% ethanol for 10 min at 4 °C and blocked with 1% BSA. Slides were incubated with an anti-α-tubulin antibody at 4 °C overnight and then incubated with an Alexa Fluor 488 secondary antibody for 2 h in dark. Slides were then mounted with a mounting solution containing DAPI. To analyze the mitotic index and proportion of cells undergoing cytokinesis, randomly selected at least six fields for each slide with a total of 700–800 cells were included for analysis. Cells undergoing mitosis were identified by the typical mitotic spindle morphology of α-tubulin that indicates prominent microtubule assembly during mitosis. Cells undergoing cytokinesis were identified by the barrel-shaped midzone microtubule bundles between two daughter cells. The mitotic index was calculated by the number of cells undergoing mitosis divided by that of total cells counted. The percentage of cytokinesis was evaluated likewise.

### 2.7 Statistical analysis

Data were represented as mean ± standard error of the mean (SEM) from at least 3 independent tests and subjected to two-tailed, unpaired Student’s *t* test using Prism Graphpad 6.0c. Difference with *p* values less than 0.05 was considered statistically significant.

## 3 Results

### 3.1 STYK1/NOK is involved in the regulation of mitosis and cytokinesis

**Figure 1:**
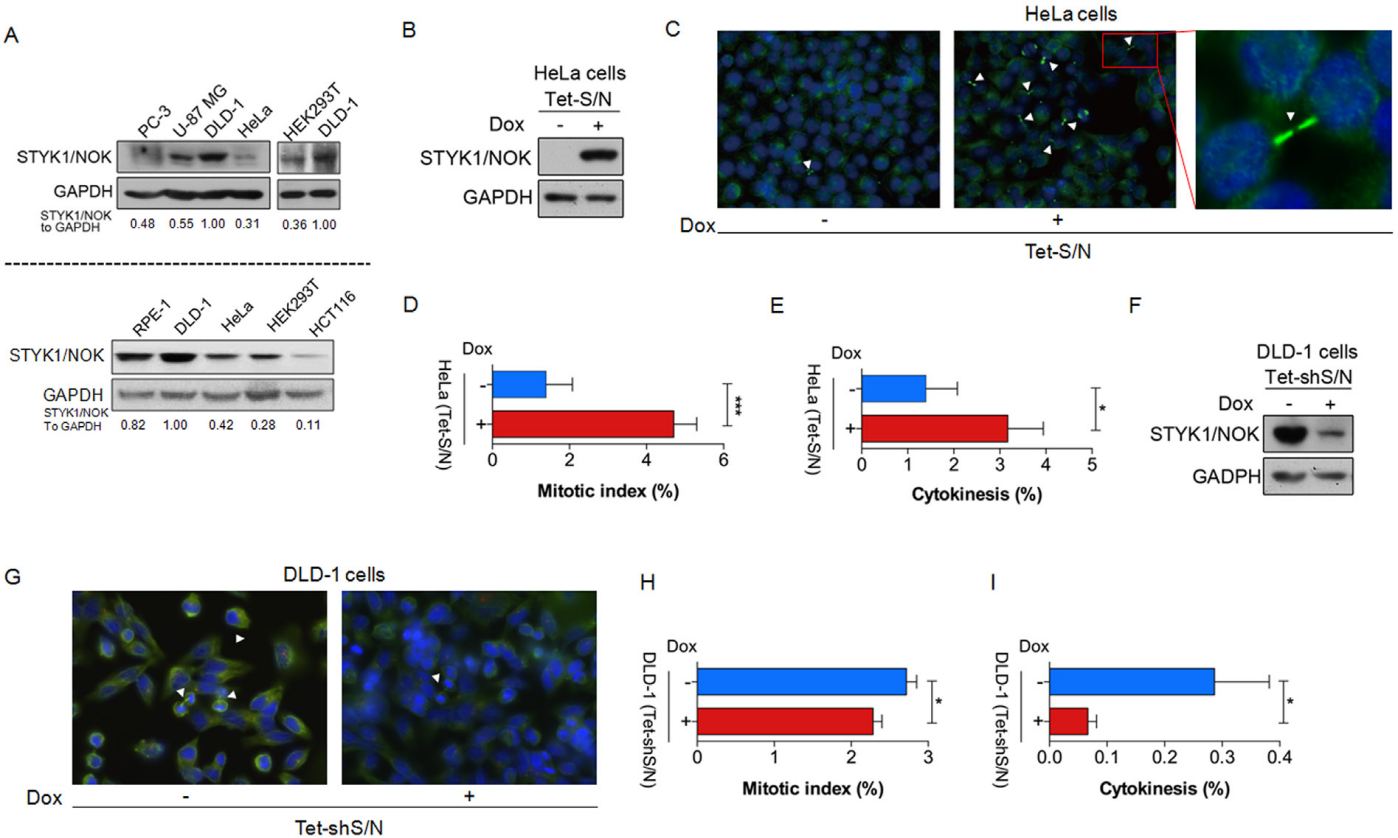
Over-expression of STYK1/NOK causes mitotic abnormality. (A) The endogenous STYK1/NOK expressions in different cell lines by immunoblot analysis in PC-3 cells, U-87 MG cells, DLD-1 cells, HeLa cells, HEK293T cells, RPE-1 cells and HCT116 cells. GAPDH was used as a loading control. The band intensities of STYK1/NOK were quantified with FIJI software and then normalized to that of GAPDH. The relative expressions of all others were compared to that of DLD-1 cells of which was set as 1.00. The original blots are presented in Supplementary Figure 1A and 1B. (B) Establishment of the doxycycline-controlled STYK1/NOK stable cell line. HeLa cells were infected with lentiviral constructs containing doxycycline-inducible STYK1/NOK expression cassettes. The infected cells were selected with 1 μg/mL of puromycin for 5 days to obtain the stable cell line Tet-S/N. Tet-S/N cells were treated with doxycycline for 48 h before harvesting. The original blots are presented in Supplementary Figure 1C. (C) Representative immunofluorescent images of the established stable HeLa cells that were stained with α-tubulin (Green) and DAPI (Blue). Arrows indicated cells undergoing cytokinesis. Quantification of immunofluorescent results presented in (C) by calculating proportions of mitotic cells (D) and cells undergoing cytokinesis (E) to the total cells counted. The data were mean ± SEM from four independent experiments. (F) Immunoblot analysis on doxycycline-induced STYK1 silencing. DLD-1 cells were stably transfected with lentiviral doxycycline-controlled lentiviral constructs expressing shSTYK1/NOK to establish the Tet-shS/N cell line. The transfected cells were selected with 2 μg/mL of puromycin for 5 days. Cells were cultured in the presence and absence of doxycycline for 3 days before harvesting. The original blots are presented in Supplementary Figure 1D. (G) Representative immunofluorescent images of the established stable Tet-shS/N cells that were stained with α-tubulin (Green) and DAPI (Blue). Quantification of immunofluorescent results showed the proportions of mitotic cells (H) and cytokinesis (I) as mean ± SEM from three independent experiments. Mitotic cells were distinguished and counted by morphological evaluation based on the α-tubulin and DAPI staining. ∗*p* < 0.05; ∗∗∗*p* < 0.001.

STYK1/NOK is an oncogene that can promote tumorigenesis and metastasis [20]. However, the effect of STYK1/NOK on cell cycle progression remains unclear. The expression levels of endogenous STYK1/NOK were first checked in a panel of human cell lines including human prostate adenocarcinoma (PC-3) cells, human brain glioblastoma (U-87 MG) cells, human colorectal adenocarcinoma (DLD-1) cells and human cervical carcinoma (HeLa) cells, as well as in two non-transformed human cell lines such as retinal pigment epithelial RPE-1 cells and human embryonic kidney 293T (HEK293T) cells. Among all, DLD-1 cells displayed the highest level of endogenous STYK1/NOK expression, while HeLa was among those with the lowest expression levels (Figure 1A upper and lower panels). Hence, HeLa cells were chosen for the construction of tetracycline-induced ectopic expression of STYK1/NOK (Tet-S/N, Figure 1B). Immunostaining analysis with an anti-α tubulin antibody and DAPI (Figure 1C) demonstrated that both mitotic index and the proportion of cells undergoing cytokinesis were significantly increased after the addition of tetracycline (Figures 1D and 1E), indicating that STYK1/NOK was involved in the regulation of mitosis and cytokinesis.

### 3.2 Knockdown of endogenous STYK1/NOK prevents cytokinesis blockage

To test the effect of STYK1/NOK depletion on mitosis and cytokinesis, we established a STYK1/NOK knockdown system (Tet-shS/N) in DLD-1 cells. After the induction of STYK1/NOK-specific RNA interference with doxycycline, the expressions of STYK1/NOK in DLD-1 cells was significantly suppressed as compared with that of the control cells (Figure 1F). As a result, the number of cells undergoing mitosis and cytokinesis was reduced, as shown by immunofluorescence with anti-α tubulin and DAPI stainings (Figure 1G). Further analysis indicated that knockdown of STYK1/NOK in DLD-1 cells significantly reduced the mitotic index (Figure 1H) and decreased the proportion of cells undergoing cytokinesis (Figure 1I). These data suggested that in cancer cells mitotic arrest and cytokinesis blockage induced by high expression of STYK1/NOK could be reversed by depletion of STYK1/NOK.

### 3.3 STYK1/NOK exhibits a cell-cycle regulated expression pattern

**Figure 2:**
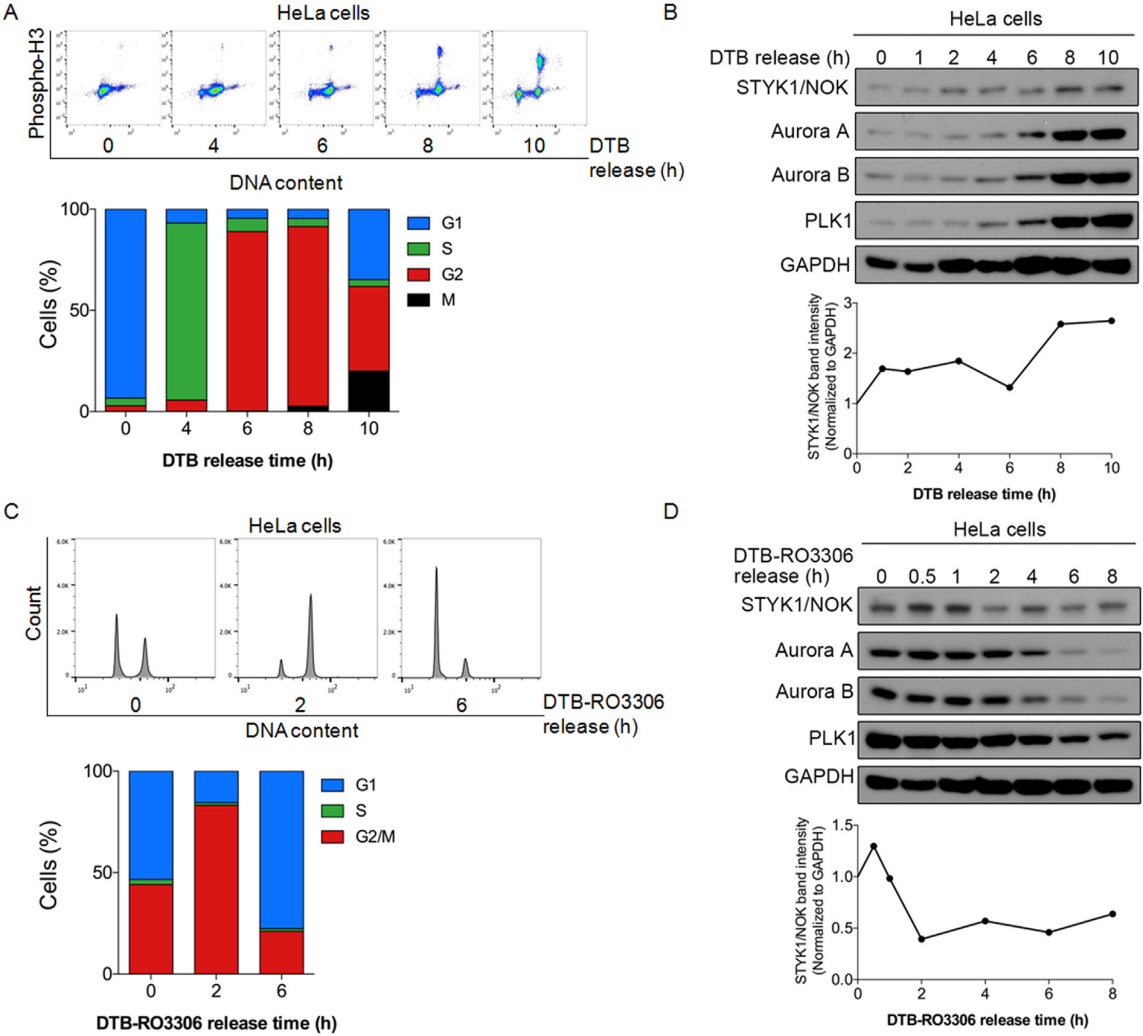
STYK1/NOK exhibits varied expression patterns during the cell cycle progression. HeLa cells were synchronized at G1/S boundary (A and B) or G2/M boundary (C and D) by double thymidine block (DTB) or DTB coupled with a CDK1 inhibitor, RO3306 (DTB-RO3306), respectively. (A) Flow cytometric analysis (upper panel) and quantitative measurements (lower panel) of the distribution of each cell cycle phase through DAPI and anti phospho-histone H3 (Ser10) staining at each time point following thymidine release. (B) Immunoblot analysis (upper panel) on the expressions of STYK1/NOK, aurora A, aurora B and PLK1 at each time point following thymidine release. The original blots are presented in Supplementary Figure 2A. GAPDH served as an internal loading control, the STYK1/NOK intensity was normalized to that of GAPDH and all time points were relative to time 0 h (lower panel). (C) Flow cytometric analysis (upper panel) and quantitative measurements (lower panel) of the distribution of each cell cycle phase through DAPI staining at each time point following RO3306 release. (D) Immunoblot analysis (upper panel) on the expressions of STYK1/NOK, aurora A, aurora B and PLK1 proteins at each time point following RO3306 release. The original blots are presented in Supplementary Figure 2B. GAPDH was used as a loading control. The STYK1/NOK intensity was normalized to that of GAPDH and all time points were relative to that of time 0 h (lower panel). The data were representative of at least two independent experiments with similar trends.

To further explore the role of STYK1/NOK in cell cycle regulation, HeLa cells were first synchronized with double thymidine block (DTB) to arrest cells at the G1/S boundary, and subsequently released to allow progression through S, G2 and M phases. Figure 2A showed that over 80% cells simultaneously progressed to G2 phase within 6 h after DTB release, and M-phase cells started to accumulate from 8 h and reached about 20% by 10 h. The expression levels of STYK1/NOK were gradually accumulated as cells entered M phase, in an analogous kinetics to those of typical mitotic kinases such as aurora A, aurora B and PLK1 (Figure 2B upper panel). Quantification and normalization of STYK1/NOK was performed to determine the expression pattern of STYK1/MOK (Figure 2B lower panel). These data suggested that STYK1/NOK was a cell-cycle regulated protein. To obtain a more complete understanding of the expression pattern of STYK1/NOK during the cell cycle, HeLa cells were synchronized with DTB followed by the addition of RO3306, a CDK1 inhibitor that blocks cells at the G2/M boundary. Figure 2C displayed that more than 80% cells simultaneously progressed to G2/M phase 2 h post RO3306 release and nearly 80% cells simultaneously entered G1 phase by 6 h upon RO3306 release. The expression levels of STYK1/NOK remained relatively high within 1 h upon RO3306 release, but subsequently decreased likely due to mitotic exit (Figure 2D). Consistently, the expression of STYK1/NOK displayed a similar cell cycle-related kinetics as that of aurora A, aurora B and PLK1, suggesting that they may share upstream regulators during cell cycle progression.

### 3.4 APC3 is an essential regulator of STYK1/NOK protein degradation

**Figure 3:**
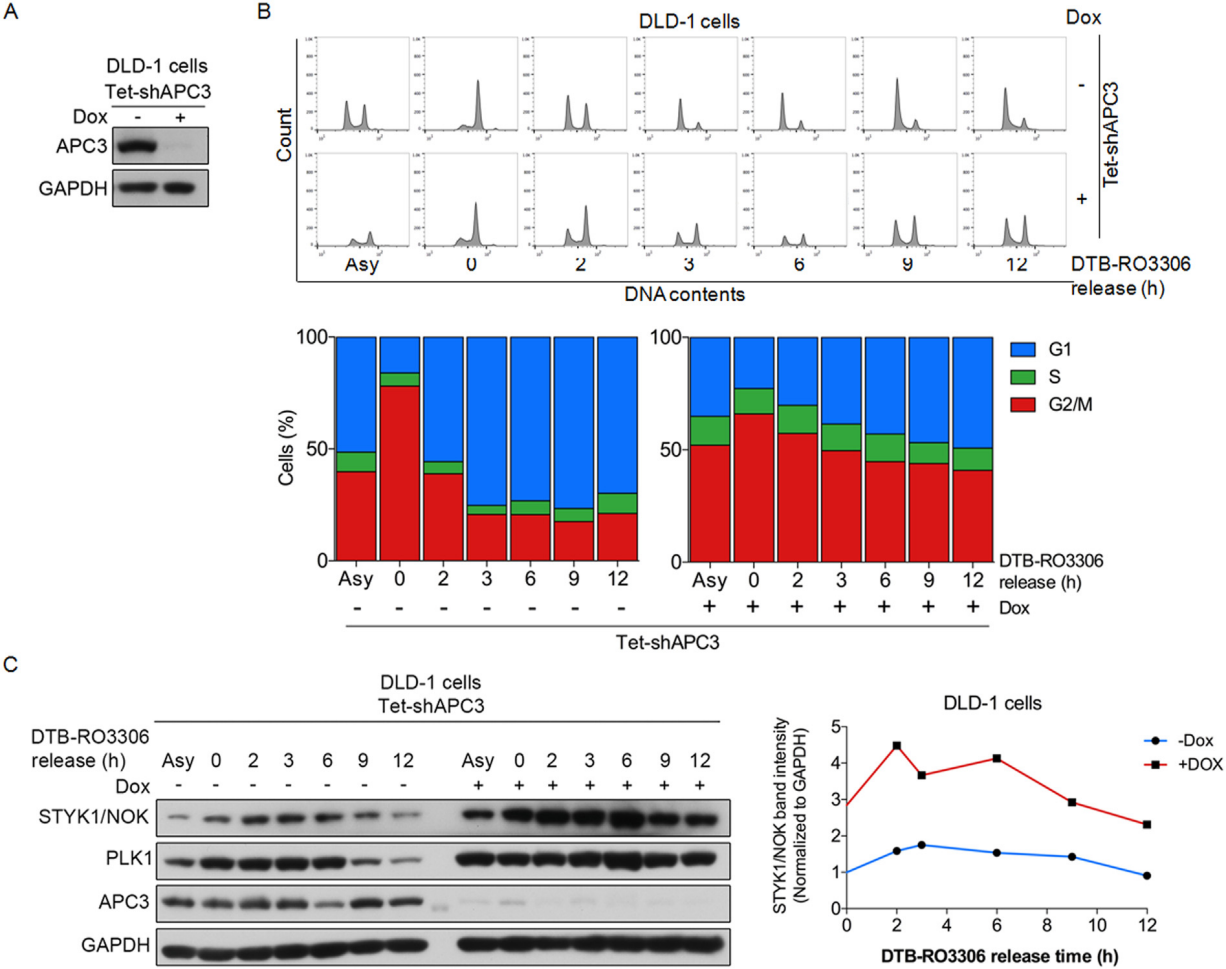
Depletion of APC3 leads to aberrant accumulation of STYK1/NOK and cell cycle arrest at G2/M. DLD-1 cells were infected with recombinant lentiviruses containing doxycycline-controlled shAPC3. The infected cells were selected with 2 μg/mL of puromycin for 5 days, then synchronized at the G2/M border with DTB-RO3306. Cells were treated with or without doxycycline for 3 days before harvesting. (A) Immunoblotting analysis of APC3 expressions in established doxycycline-controlled shAPC3 stable DLD-1 cell line. GAPDH served as an internal control. The original blots are presented in Supplementary Figure 3A. (B) Flow cytometric analysis (upper panel) and quantitative measurements (lower panel) of the distribution of each cell cycle phase through DAPI staining at each time point following RO3306 release with or without doxycycline. (C) Immunoblotting analysis (left panel) of the expressions of STYK1/NOK, PLK1, and APC3 proteins at each time point following RO3306 release as described in (B). The original blots are presented in Supplementary Figure 3B. The expressions of GAPDH served as an internal control. The STYK1/NOK intensity was normalized to that of GAPDH and all time points were relative to time 0 h without doxycycline (right panel).

Ubiquitin-mediated protein degradation by anaphase-promoting complex (APC/C) targets a large number of mitotic kinases including aurora kinases A and B, and all these proteins are important regulators for anaphase onset and mitotic exit [21]. Depleting APC3, the key component of APC/C, causes the delayed clearance of its substrates and failure to complete mitosis and cytokinesis [22]. Since STYK1/NOK shares a similar expression pattern with these mitotic protein kinases (Figures 2B and 2D), we hypothesized that STYK1/NOK may also be regulated by key components of APC/C. To test this hypothesis, we first introduced tetracycline-inducible shRNAs specially targeting APC3 (shAPC3) in DLD-1 cells (Tet-shAPC3) and confirmed the reduction of APC3 expression in the presence of doxycycline (Figure 3A). The Tet-shAPC3 cells were synchronized at the G2/M border by DTB coupled with RO3306 as described earlier. The synchronized cells were subsequently released in the presence and absence of doxycycline for the indicated intervals. Flow cytometric analysis revealed that knocking down APC3 caused G2/M arrest (Figure 3B). Western blotting analysis further demonstrated that depletion of APC3 by shRNA led to significantly enhanced expressions of both STYK1/NOK and PLK1 (Figure 3C), which correlated with G2/M retention (Figure 3B). The expression of STYK1/NOK in DLD-1 cells displayed a higher level in mitosis and declined when mitosis exited (Figure 3C). These data suggest that STYK1/NOK, like other mitotic kinases, might also be regulated by the APC/C complex.

### 3.5 CDH1 interacts with STYK1/NOK and affect its protein stability

**Figure 4:**
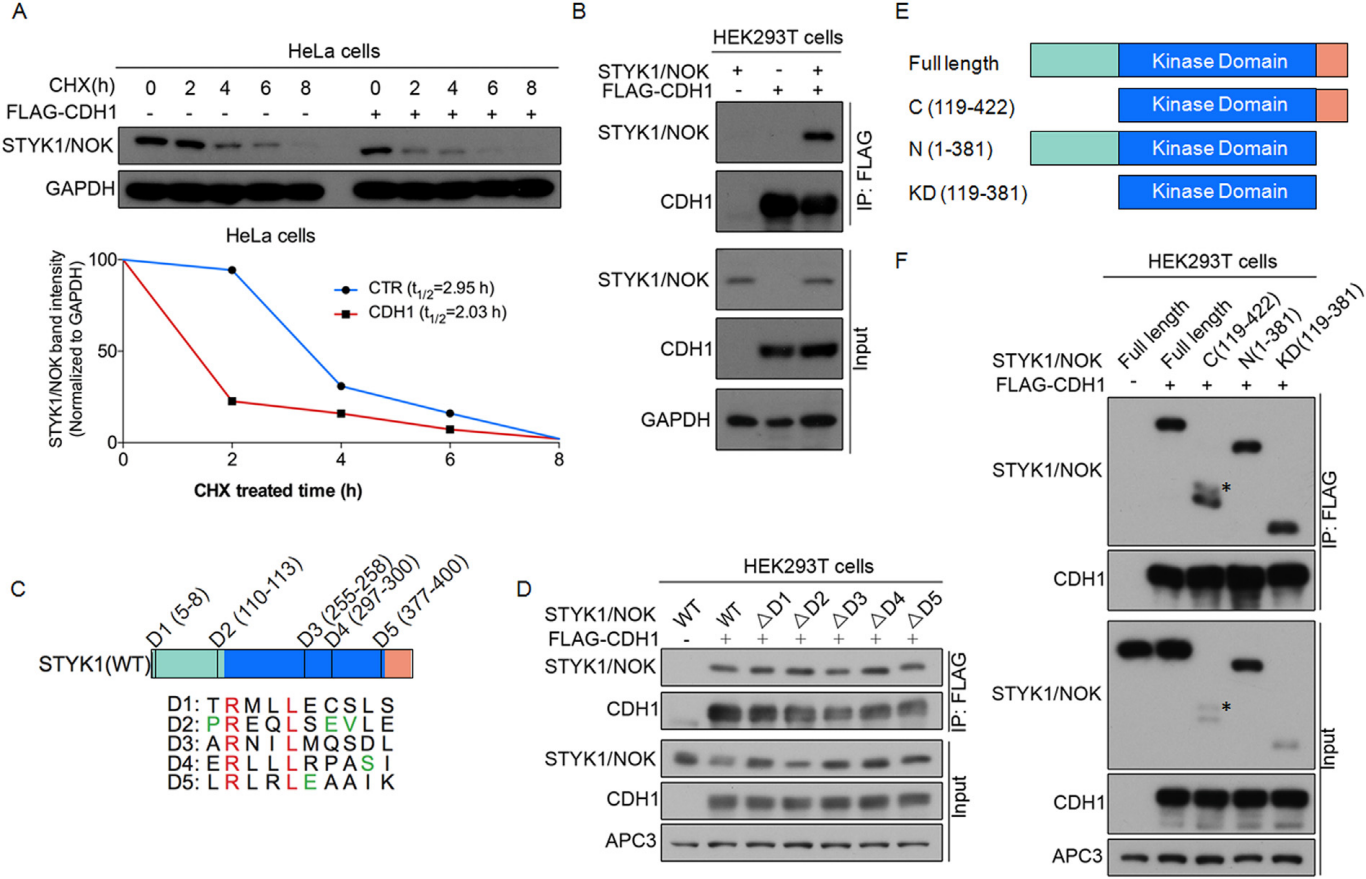
CDH1 interacts with STYK1/NOK in a kinase domain dependent manner. (A) HeLa cells were co-transfected with pcDNA3.0-STYK1/NOK and FLAG-CDH1 or an empty vector. Cells were treated with 50 μg/mL of cycloheximide for the indicated time before harvesting. Immunoblot analysis (upper panel) was conducted to detect the expression of STYK1/NOK. The expression of GAPDH served as an internal control. The original blots are presented in Supplementary Figure 4A. Quantification analysis (lower panel) of the relative band intensity of STYK1/NOK was performed to determine the protein half-life of STYK1/NOK. The STYK1/NOK band intensities were normalized to that of GAPDH, then further normalized to that of t = 0 time point. (B) HEK293T cells were co-transfected with pcDNA3.0-STYK1/NOK and FLAG-CDH1 and treated with 10 μM of MG132 for 4 h before harvesting. Co-immunoprecipitation for detection of STYK1/NOK and CDH1 interaction was then performed. The original blots are presented in Supplementary Figure 4B. (C) Schematic representation of the wild type STYK1/NOK domain structure and its five D-boxes (D1 to D5). Red letters represent conserved residues and green letters represent preferred residues. (D) HEK293T cells were co-transfected with FLAG-CDH1 and pcDNA3.0-STYK1/NOK or its D-box deleted mutants (ΔD1 to ΔD5) and treated with 10 μM of MG132 for 4 h before harvesting. Coimmunoprecipitation was carried out to detect the interaction between CDH1 and either STYK1/NOK or its mutants. The original blots are presented in Supplementary Figure 4C. (E) Schematic presentation of the full-length STYK1/NOK and its truncated forms. (F) HEK293T cells were co-transfected with FLAG-CDH1 and pcDNA3.0-STYK1/NOK or its truncated forms and treated with 10 μM of MG132 for 4 h before harvesting. Coimmunoprecipitation assay was used to detect the interaction between CDH1 and full-length STYK1/NOK or its truncated forms. The star ∗ represents non-specific proteins. The original blots are presented in Supplementary Figure 4D.

Substrates of APC/C complex are usually recruited by its coactivators such as CDC20 and CDH1. CDC20 works at S phase and early mitosis [6], while CDH1 mainly functions in both late mitosis and G1 phase [23]. We hypothesized that CDH1 is likely a major regulator of STYK1/NOK. To test this hypothesis, we ectopically expressed CDH1 in HeLa cells in the presence of cycloheximide to assess the effects of CDH1 on protein turnover of ectopically expressed STYK1/NOK. Western blotting analysis demonstrated that CDH1 facilitated the degradation of STYK1/NOK (Figure 4A upper panel), manifested by a reduced half-life of STYK1/NOK protein (Figure 4A lower panel). To further determine whether STYK1/NOK could physically interact with CDH1, we performed a co-immunoprecipitation assay by co-expressing STYK1/NOK and FLAG-tagged CDH1 in HEK293T cells. Results in Figure 4B demonstrated that STYK1/NOK could be immunoprecipitated by anti-FLAG antibody, indicating that STYK1/NOK physically interacted with CDH1.

### 3.6 STYK1/NOK binds to CDH1 in a kinase-domain dependent manner

CDH1 usually recognizes substrates that contain degradation signals conferred by D-boxes or KEN-boxes [24]. Sequence analysis uncovered five D-boxes in STYK1/NOK protein and among them, D2, D4 and D5 contain conserved residues [4] (as highlighted in Figure 4C). In order to assess whether the interaction between CDH1 and STYK1/NOK was dependent on these D-boxes, we mutated all five D boxes individually in STYK1/NOK by deleting the corresponding RXXL residues. The co-immunoprecipitation results showed that none of the D-boxes mutants impaired the interactions between CDH1 and STYK1/NOK (Figure 4D). The next question that we attempted to ask was which domain of STYK1/NOK was responsible for the interaction with CDH1. To address this issue directly, we constructed three truncation forms of STYK1/NOK as depicted in Figure 4E. The results of co-immunoprecipitation showed that except the kinase domain, removal of the N terminus, C terminus, or both termini of STYK1/NOK did not interrupt the interaction between STYK1 and CDH1 (Figure 4F). Thus, the results indicated that the kinase domain of STYK1/NOK was the main region in charge of its interaction with CDH1.

## 4 Discussion

STYK1/NOK plays an important role in promoting tumorigenesis, invasion and metastasis [20]. However, the underlying mechanism is largely unexplored. Our current report provides evidence to show that STYK1/NOK may have a direct impact on the maintenance of mitotic fidelity during cell division by directly interacting with CDH1 at late mitosis.

APC/C complex plays a vital role to ensure the precise control of cell cycle transition through mitosis. The binding with the coactivators is required not only for APC/C-mediated substrate recognition and recruitment in D-box/KEN-box dependent manner, but also for the enzymatic function of APC/C as an E3 ligase [2]. However, studies also demonstrate that substrate recognition may be D-box and KEN-box independent. Castro et al. found that CDH1 recognized substrate Xkid via GxEN but not D-box [25]. GxEN is a transposable degradation signal that may function as a D-box or KEN box like signal to promote the degradation of the targeted proteins. However, not all degrons of the identified substrates have been clearly defined [26]. In this study, we found that although there are five D boxes in STYK1/NOK, none of them could have the canonical D-box function as those found in cyclin B1 [27] and PLK1 [28]. Our data indicate that APC/C may influence STYK1/NOK expression in a D-box-independent manner. Deletion study showed that neither the N terminus or the C terminus is essential for the interaction between STYK1/NOK and CDH1, suggesting that the kinase domain of STYK1/NOK was sufficient for its interaction with CDH1. However, further efforts are needed to identify a specific motif that mediates the interaction of STYK1/NOK with CDH1.

The APC/C complex mediates the degradation of its substrates, which is tightly regulated to ensure accurate separation of sister chromatids during mitosis. Any abnormality during this process can cause chromosome missegregation, formation of anaphase bridges and micronuclei, cytokinesis failure and polyploidy [29], all contributing to tumorigenesis. In current study, we found that ectopic expression of STYK1/NOK could induce mitotic arrest and aberrant cytokinesis, whereas knockdown of STYK1/NOK could alleviate the abnormal mitotic phenotypes. STYK1/NOK functions as an oncogene since tumorigenesis can be induced when BaF3-NOK were subcutaneously injected into nude mice [12]. However, little is known about how STYK1/NOK promotes tumorigenesis and whether deregulation of STYK1/NOK may impair the fidelity of genetic transmission during the cell cycle. Our study lends credence to the notion that indeed, STYK1/NOK is regulated during cell division and that deregulation of STYK1/NOK affects the central machinery of genetic transmission during mitosis, which often confers vulnerability to neoplastic transformation and tumor formation. Our data also demonstrate that blocking STYK1/NOK ameliorates mitotic defects, suggesting that therapeutic targeting STYK1/NOK may represent a novel approach in preventing STYK1/NOK-associated tumor progression and metastasis.

## Declarations

### Author contribution statement

Su-Lin Zeng: Conceived and designed the experiments; Performed the experiments; Analyzed and interpreted the data; Wrote the first draft of the paper.

Suraj Patel, Mo-Qi Lv, Daphne Zhu: Performed the experiments.

Wen H Shen: Conceived and designed the experiments; Contributed reagents, materials, analysis tools or data; Wrote the paper.

Li Liu: Conceived the experiments; Analyzed and interpreted the data; Contributed reagents, materials, analysis tools or data; Re-wrote the paper and revised the paper.

### Funding statement

Professor Li Liu was supported by 10.13039/501100011176Chinese Academy of Medical Sciences Initiative for Innovative Medicine (2017-I2M-3-007).

Dr. Su-Lin Zeng was supported by Chinese Scholarship Council.

Professor Wen H Shen was supported by T. Hirschl/Monique Weill-Caulier Trust.

### Data availability statement

Data included in article/supp. material/referenced in article.

### Declaration of interest’s statement

The authors declare no conflict of interest.

### Additional information

No additional information is available for this paper.
