# Supplementary material for: STYK1/NOK affects cell cycle late mitosis and directly interacts with anaphase-promoting complex activator CDH1
Source: Heliyon. 2022 Dec 5;8(12):e12058. doi: 10.1016/j.heliyon.2022.e12058 (PMC9732331; doi:10.1016/j.heliyon.2022.e12058)
Supplement: Supplementary data-11222022 [file mmc1.docx]

**Supplementary data**

The original data of Fig. 1A

STYK1/NOK:
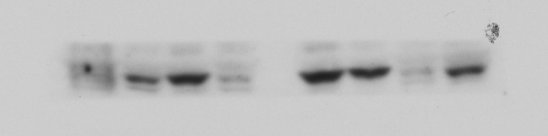


GAPDH:
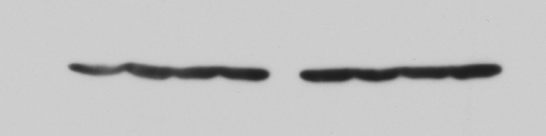


Samples: PC-3, U-87 MG, DLD-1, HeLa

The original data of Fig. 1B

STYK1/NOK:
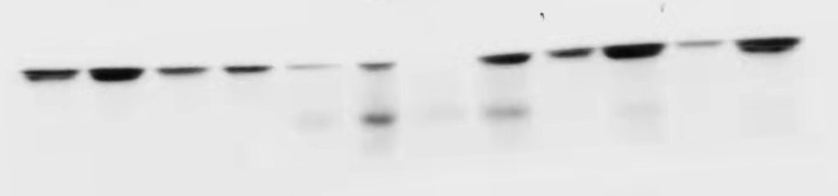


GAPDH:
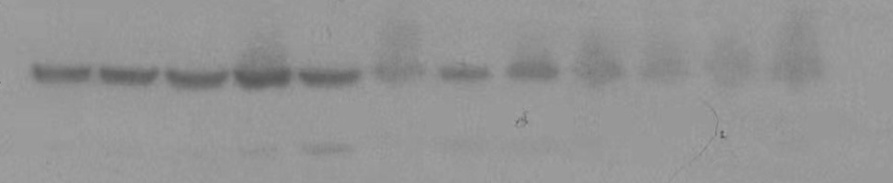


Samples: RPE-1, DLD-1, HeLa, HEK293T, HCT116

The original data of Fig. 1C

STYK1/NOK:
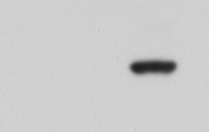


GAPDH:
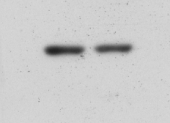


Samples: Tet-S/N HeLa cells without (-) Dox, Tet-S/N cells with (+) Dox

The original data of Fig. 1D

STYK1/NOK:
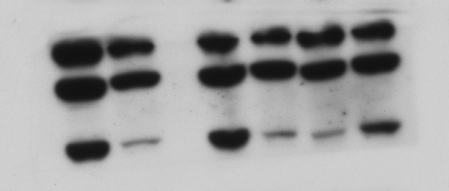


GAPDH:
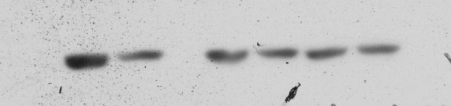


Samples: Tet-shS/N DLD-1 cells without (-) Dox; Tet-shS/N cells with (+) Dox

The original data of Fig. 2A

STYK1/NOK:
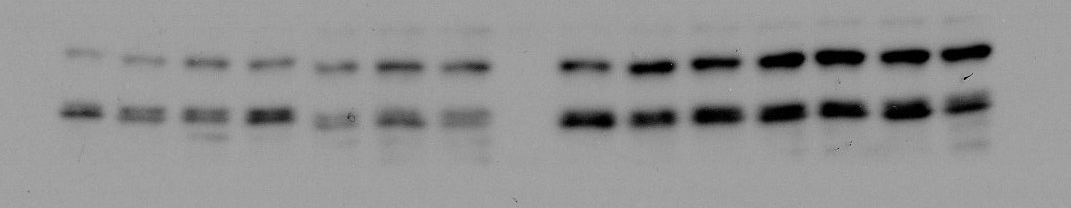


Aurora A:
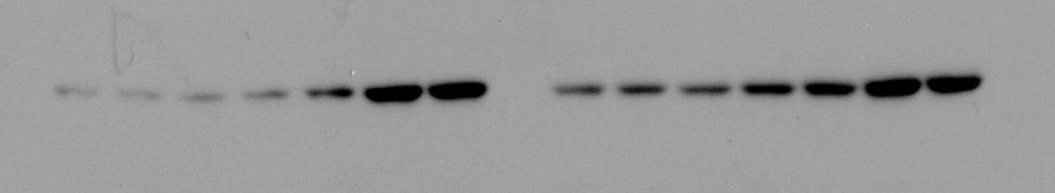


Aurora B:
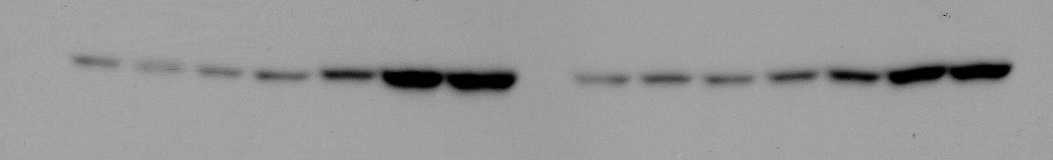


PLK1:
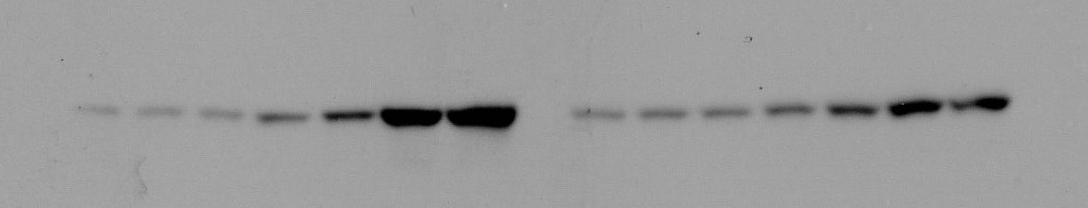


GAPDH:
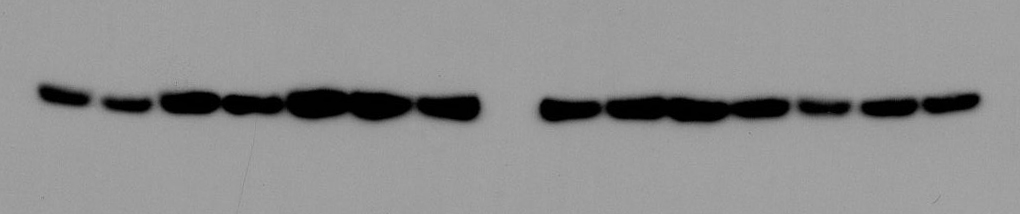


Samples: DTB release (h) 0, 1, 2, 4, 6, 8, 10

The original data of Fig. 2B

STYK1/NOK:
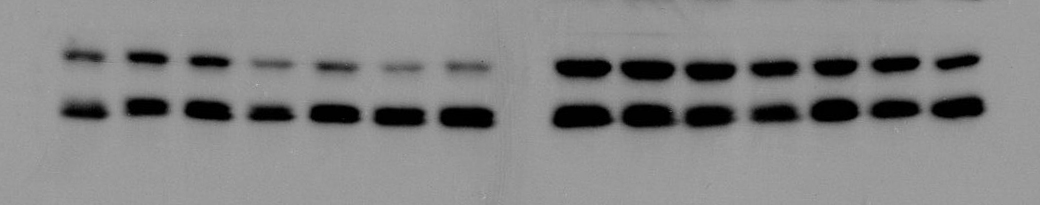


Aurora A:
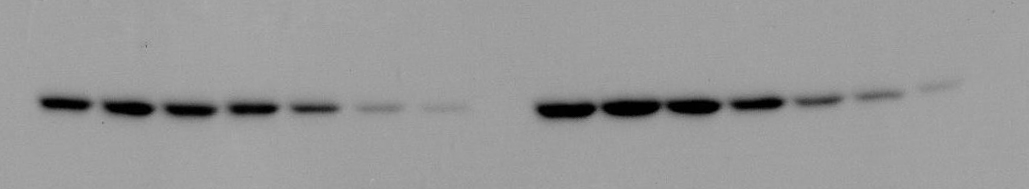


Aurora B:
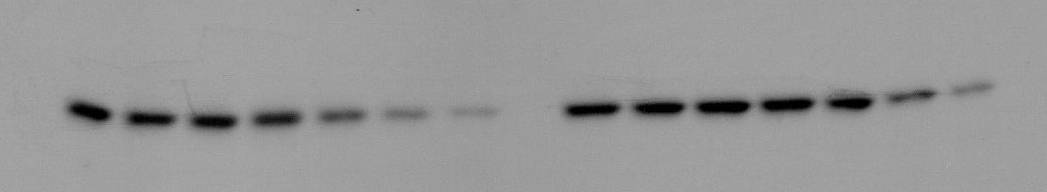


PLK1:
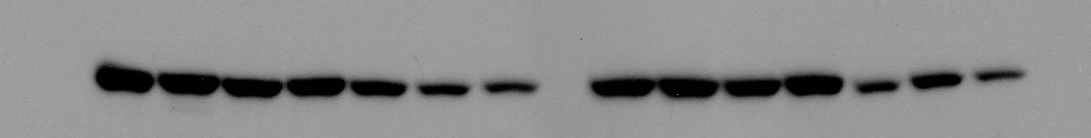


GAPDH:
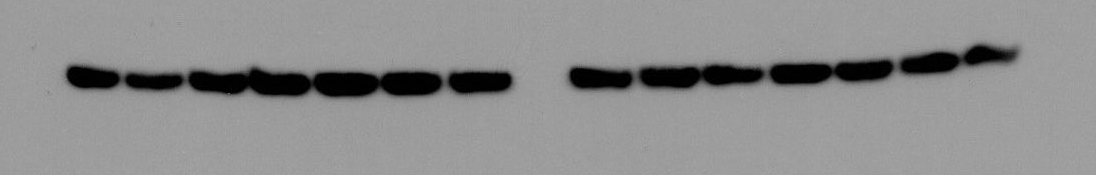


Samples: DTB-RO3306 release (h) 0, 0.5, 1, 2, 4, 6, 8

The original data of Fig. 3A

APC3:
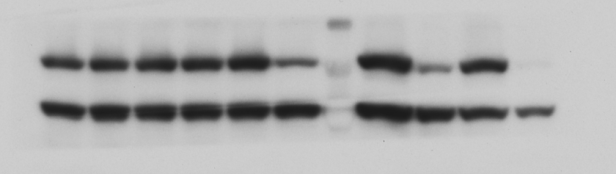


GAPDH:
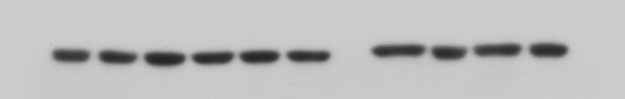


Samples: Tet-shAPC3 DLD-1 cells without (-) Dox; Tet-shAPC3 DLD-1 cells with (+) Dox

The original data of Fig. 3B

STYK1/NOK:
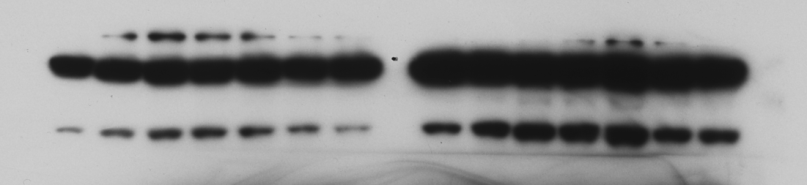


PLK1:
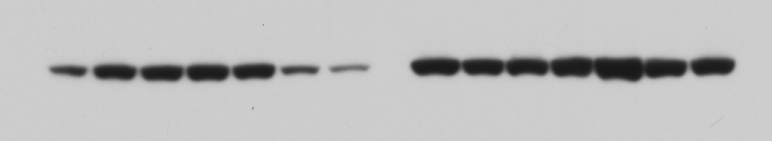


APC3:
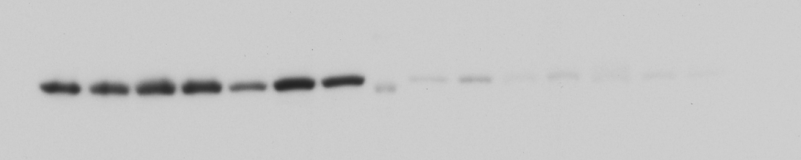


GAPDH:
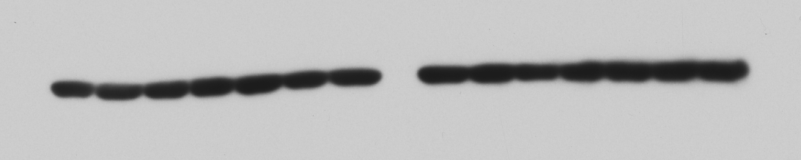


Samples: Tet-shAPC3 DLD-1 cells without (-) Dox Asy, DTB-RO3306 release (h) 0, 2, 3, 6, 9, 12; Tet-shAPC3 DLD-1 cells with (+) Dox Asy, DTB-RO3306 release (h) 0, 2, 3, 6, 9, 12

The original data of Fig. 4A

STYK1/NOK:
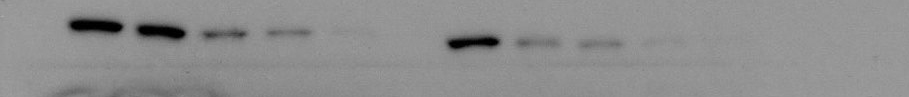


GAPDH:
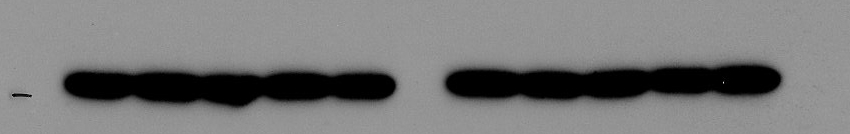


Samples: without CDH1 CHX (h) 0, 2, 4, 6, 8; with CDH1 CHX (h) 0, 2, 4, 6, 8

The original data of Fig. 4B

STYK1/NOK:
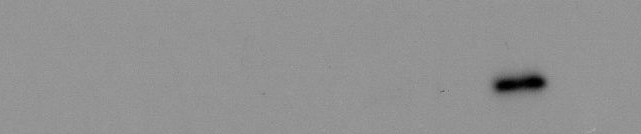


Samples: IP: FLAG

STYK1/NOK (+) FLAG-CDH1(-), STYK1/NOK (-) CDH1(+), STYK1/NOK (+) CDH1(+)

STYK1/NOK:
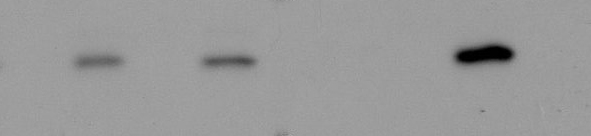


GAPDH:
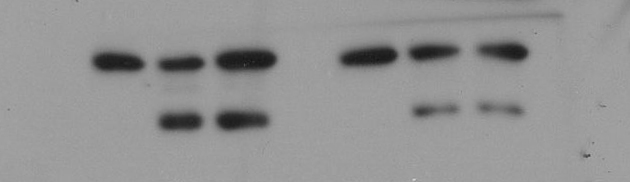


Samples: Input

STYK1/NOK (+) FLAG-CDH1(-), STYK1/NOK (-) CDH1(+), STYK1/NOK (+) CDH1(+)

CDH1:
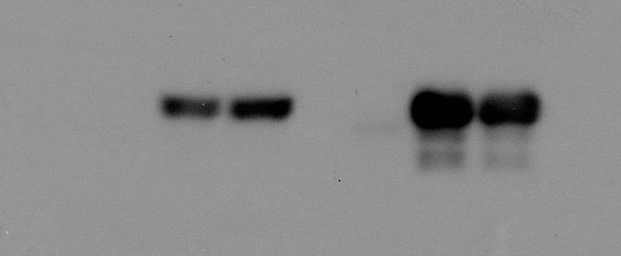


Samples: Input-left box

STYK1/NOK (+) FLAG-CDH1(-), STYK1/NOK (-) CDH1(+), STYK1/NOK (+) CDH1(+);

IP: FLAG-right box

STYK1/NOK (+) FLAG-CDH1(-), STYK1/NOK (-) CDH1(+), STYK1/NOK (+) CDH1(+)

The original data of Fig. 4C

STYK1/NOK:
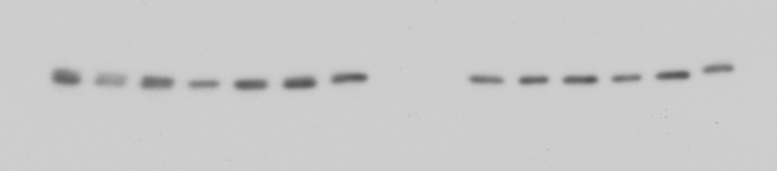


CDH1:
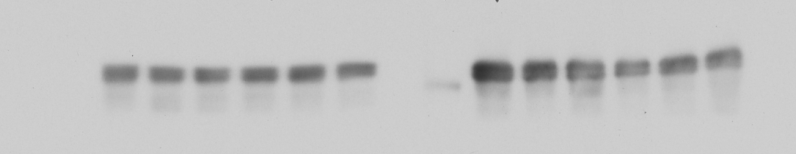


Samples: Input-left box

WT (+) FLAG-CDH1(-), WT (+) CDH1 (+), ΔD1 (+) CDH1 (+), ΔD2 (+) CDH1 (+), ΔD3 (+) CDH1 (+), ΔD4 (+) CDH1 (+), ΔD5 (+) CDH1 (+);

IP: FLAG-right box

WT (+) FLAG-CDH1(-), WT (+) CDH1 (+), ΔD1 (+) CDH1 (+), ΔD2 (+) CDH1 (+), ΔD3 (+) CDH1 (+), ΔD4 (+) CDH1 (+), ΔD5 (+) CDH1 (+);

APC3:
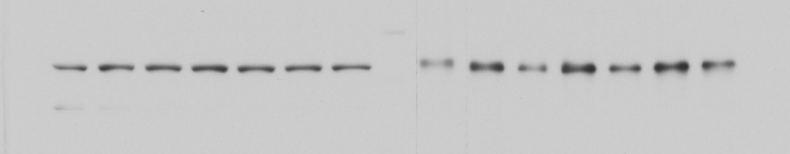


Samples: Input

WT (+) CDH1 (-), WT (+) CDH1 (+), ΔD1 (+) CDH1 (+), ΔD2 (+) CDH1 (+), ΔD3 (+) CDH1 (+), ΔD4 (+) CDH1 (+), ΔD5 (+) CDH1 (+)

The original data of Fig. 4D

STYK1:
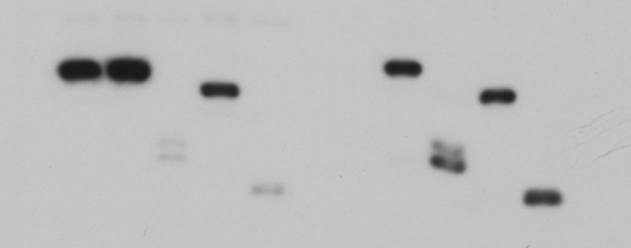


CDH1:
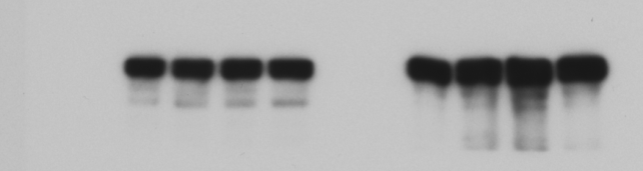


Samples: Input-left box

Full length (+) FLAG-CDH1 (-), Full length (+) CDH1 (+), C (119-422) (+) CDH1 (+), N (1-381) (+) CDH1 (+), KD (119-381) (+) CDH1 (+)

IP: FLAG-right box

Full length (+) FLAG-CDH1 (-), Full length (+) CDH1 (+), C (119-422) (+) CDH1 (+), N (1-381) (+) CDH1 (+), KD (119-381) (+) CDH1 (+)

APC3:
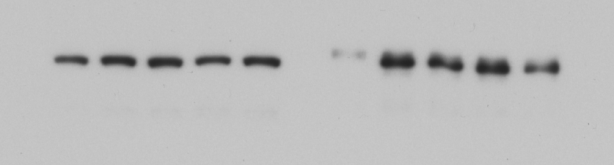


Samples: Input

Full length (+) FLAG-CDH1 (-), Full length (+) CDH1 (+), C (119-422) (+) CDH1 (+), N (1-381) (+) CDH1 (+), KD (119-381) (+) CDH1 (+)
